# Promise to Practice: Reimagining Artificial Intelligence for Equitable Global Health Impact

**DOI:** 10.5334/aogh.5268

**Published:** 2026-06-03

**Authors:** Shama Patel, Katherine O. Robsky, Wenhui Mao, Umar Usman, Lorena Garcia, Olubukola Omobowale, Gugu Mchunu, Joowhan Sung, Gari Clifford, Anant Madabhushi, Azra Ismail, Saara Bidiwala, Seemab Mehmood, Christine Ngaruiya, Paul E. Kilgore, Keith Martin

**Affiliations:** 1 Brown University, Division of Global Emergency Medicine, Adjunct Faculty, Providence, RI, USA; 2 Africa Health Sciences University, Division of Emergency Medicine, Adjunct Faculty, Kigali, Rwanda; 3 Center for Global Health Practice and Impact, Georgetown University, Washington, DC, USA; 4 Duke Global Health Innovation Center, Duke University, Durham, NC, USA; 5 Duke Global Health Institute, Duke University, Durham, NC, USA; 6 AFREhealth West African Chapter, Secretariat, Kwame Nkrumah University of Science and Technology, Kumasi, Ghana; 7 University of California Davis, School of Medicine, Davis, CA, USA; 8 Rehabilitative and Social Medicine Unit, Department of Community Medicine, College of Medicine, University of Ibadan, Nigeria; 9 Durban University of Technology, Faculty of Health Sciences, South Africa; 10 Johns Hopkins University School of Medicine, Division of Infectious Diseases, Baltimore, MD, USA; 11 Emory University, Department of Biomedical Informatics, Atlanta, GA, USA; 12 Georgia Institute of Technology and Emory University, Department of Biomedical Engineering, Atlanta, GA, USA; 13 Atlanta Department of Veterans Affairs Medical Center, Atlanta, GA, USA; 14 Fatima Jinnah Medical University, Lahore, Pakistan; 15 Stanford School of Medicine, Stanford EM International Global and Population Health Section (SEMI), Department of Emergency Medicine, Palo Alto, CA, USA; 16 Wayne State University, Department of Pharmacy Practice, Eugene Applebaum College of Pharmacy and Health Sciences, and the Center for Emerging and Infectious Diseases, Detroit, Michigan, USA; 17 Consortium of Universities for Global Health, Washington, DC, USA

**Keywords:** artificial intelligence, global health, machine learning, prediction models, digital health, technology, health equity

## Abstract

Artificial Intelligence (AI) is transforming health worldwide, yet its benefits remain unevenly distributed and insufficiently evaluated in real-world settings. Drawing on a structured narrative landscape review conducted by the Consortium of Universities of Global Health Research Committee’s AI working group and deliberations from its 2025 pre-conference session (171 registrants), we synthesize AI applications across education, epidemiology, and clinical medicine, with an emphasis on data equity in global health. The review drew on peer-reviewed and gray literature across these domains and was synthesized thematically to identify implementation barriers, governance challenges, and equity implications. In conjunction with illustrative case studies, we identify five strategic imperatives: contextualized governance frameworks, equitable capacity-building, rigorous implementation and cost-effectiveness research, open knowledge repositories, and community-centered ethical design. We argue that AI must shift from technological optimism to locally led, evidence-driven, and equity-centered deployment. Without these paradigm shifts, AI risks reinforcing the very inequities it aims to solve.

## Introduction

Artificial Intelligence (AI) is increasingly positioned as a transformative tool for global health, spanning diagnostics, outbreak prediction, health systems strengthening, and medical education [1]. The World Health Organization’s Global Initiative on AI for Health recognizes the potential of AI to advance universal health coverage and health equity while cautioning against bias, governance gaps, and insufficient evaluation of real-world effectiveness [2]. Yet critical questions remain regarding access, evidence, governance, and equity [3–7]: (1) Who designs these tools, for whom, under whose oversight? (2) How do we ensure the tools are utilized responsibly and ethically? (3) What happens when technologies outpace the systems meant to govern them?

To explore these issues, the Consortium of Universities of Global Health (CUGH) Research Committee‘s AI Working Group conducted a structured narrative landscape review through a series of virtual consultative meetings, examining AI applications across education, epidemiology and surveillance, clinical medicine, and data equity (which we conceptualize as fair representation, stewardship, and use of data across populations). This review was designed as a structured rapid narrative scan rather than a formal systematic review. Working group members examined recent peer-reviewed and gray literature with attention to applications, implementation barriers, governance issues, and equity implications across the four domains. Findings were synthesized thematically and used to inform both the CUGH pre-conference session and the strategic priorities presented in this viewpoint. This work culminated in a dedicated pre-conference session titled “Harnessing Technology and AI for Collaborative Breakthroughs in Global Health” (Atlanta, February 2025). With 171 registrants and 14 speakers from academia, public health, and industry, the session featured results of our landscape review, as well as trainee perspectives, field-based case studies, and a moderated expert dialogue. This viewpoint synthesizes literature and field-based insights to articulate structural priorities for equitable AI deployment in global health.

### A landscape in motion: Current state of AI in global health

The AI working group reviewed four global health domains, education, epidemiology/surveillance, clinical medicine, and data equity, to identify major applications, stakeholders, implementation challenges, evidence gaps, and illustrative case examples. Findings revealed a wide range of AI applications, from personalized education to predictive analytics, and highlighted the fragmented nature of deployment [8–10]. Despite domain-specific differences, several common themes emerged across settings (Table 1).

**Table 1:** AI applications, evidence maturity, and equity risks across global health domains.

DOMAIN	EXAMPLE AI APPLICATIONS	EVIDENCE MATURITY	PRIMARY EQUITY RISKS	KEY GAPS
Education	Intelligent tutoring systems, adaptive learning platforms, virtual simulation, curriculum analytics and administration	Low–moderate	Digital divide, unequal access to hardware and connectivity	Faculty training, contextualized curricula, evaluation of learning outcomes
Epidemiology and surveillance	Outbreak prediction, contact tracing, pathogen modeling	Moderate	Surveillance overreach, data misuse, limited transparency	Governance frameworks, cross-border data stewardship
Clinical medicine	Diagnostic imaging, clinical decision support, telemedicine tools	Variable	Algorithmic bias, dataset mismatch, unclear accountability	Outcome-based evaluation, cost-effectiveness data
Data equity	Bias audits, fairness metrics, participatory design approaches	Emerging	Tokenistic inclusion, lack of enforcement	Regulatory authority, standardized equity benchmarks

**Education:** AI applications in global health education include adaptive learning platforms, automated feedback systems, simulation technologies, and curriculum analytics [11]. Early evidence suggests improvements in engagement and personalization, yet robust longitudinal evaluations linking AI-supported education to workforce performance or patient outcomes remain scarce [12–15]. Most published evaluations remain short-term or descriptive, leaving uncertainty about effects on workforce competency, clinical behavior, or patient outcomes. Challenges include unequal access to hardware and connectivity, limited faculty preparedness, and unresolved ethical questions around data privacy [10].

In many low- and middle-income country (LMIC) settings, AI-enhanced education risks widening disparities if infrastructure and digital literacy gaps persist. Key stakeholders include educational institutions, healthcare providers, technology developers, students, educators, policymakers, and funding agencies.

**Epidemiology and surveillance:** AI tools increasingly support outbreak detection, predictive modeling, and digital contact tracing. Platforms like BlueDot (Canada), HealthMap (Boston), Arogya Setu (India), and BioSense (US CDC) illustrate diverse deployment models. Although technical feasibility is often demonstrated, few longitudinal studies assess model degradation, false-positive burden, cross-border data governance, or comparative cost-effectiveness relative to conventional surveillance [8, 16]. Funding primarily comes from government agencies (NIH, CDC, DARPA, EU, PHAC, UKRI) and philanthropic organizations (Gates Foundation, Wellcome Trust), which may limit the long-term sustainability of these efforts.

**Clinical medicine:** AI applications include education augmentation aides, diagnostic imaging support, decision-support systems, ambient documentation tools, drug discovery, and virtual care optimization [17]. AI-assisted tools may support asynchronous learning and remote care workflows, but evidence of broad clinical benefit remains uneven across settings [11]. Additionally, ambient scribes and related tools aimed at reducing administrative and cognitive burden are being adopted with increased frequency in various clinical settings [17]. Technical accuracy often precedes implementation evidence, and generalizability remains a significant concern. Implementation challenges, including provider trust, workflow integration, and accountability frameworks, often dominate impact. Reported benefits include improved diagnostic performance in selected use cases, more efficient resource allocation, and accelerated analytic workflows; however, these gains do not consistently translate into real-world effectiveness across settings. Several highly accurate models also fail at the point of implementation because workflow disruption, poor calibration in new populations, unclear liability, or dependence on infrastructure is unavailable in low-resource environments.

**Data equity:** Across domains, concerns regarding algorithmic bias, dataset imbalance, and limited transparency persist [8]. Here, data equity includes who is represented in datasets, who governs access to those data, and who benefits from the resulting tools. AI systems may reproduce structural inequities embedded within training data and governance architectures [18, 19]. Although participatory design methods and fairness auditing frameworks are increasingly proposed, regulatory enforcement and accountability mechanisms remain inconsistent, particularly across LMIC contexts [20]. In practice, participation may remain consultative rather than power-sharing, limiting the corrective potential of these approaches.

### Conference session insights

To share the findings of the landscape review and foster dialogue on AI in global health research, a pre-conference session, “Harnessing Technology and AI for Collaborative Breakthroughs in Global Health,” was held at the 2025 CUGH conference. The session opened with four “Setting the Scene” presentations: the AI Working Group’s landscape review, results from a CUGH Trainee Advisory Committee survey of emerging global health professionals, a review on predictive AI applications in African settings, and a review of AI policies in LMICs.

Across the presentations, a consistent theme was uneven preparedness for AI adoption in global health. Trainees reported interest in formal AI education but limited confidence in current use, while examples from low-resource settings showed that implementation barriers, governance gaps, and infrastructure constraints often outweighed technical promise. These observations reinforced the need for workforce development, context-sensitive tool selection, cross-industry education resource development, and a stronger policy and governance framework to mitigate the potential of AI to reinforce, perpetuate, and embed inequities.

### Innovation in practice: Case studies across contexts

The session’s second part featured five case studies that moved the discussion from principle to practice (Table 2). For example, a study from Uganda showed that imaging-based diagnostic support may perform well analytically yet remain constrained by electricity reliability, device maintenance, connectivity, and local workflow integration. Similarly, AI-enabled cancer screening across multiple LMIC settings highlighted the dependence of image-classification tools on large, well-annotated, and locally representative datasets. Additionally, AI-powered systems illustrated potential in improving healthcare service delivery and clinical decision support. Across cases, the central lesson was that contextual readiness, governance, and implementation feasibility often determine impact more than model performance alone.

**Table 2:** Illustrative AI case studies presented at the CUGH 2025 pre-conference session.

USE CASE	GEOGRAPHIC CONTEXT	AI FUNCTION	PRIMARY BARRIER IDENTIFIED	KEY LESSON
Cancer screening	Multiple LMIC settings	Image classification	Limited annotated datasets	Data inequity constrains scalability
Mobile health applications	Multiple regions	Health service access optimization	Governance and regulation	Policy frameworks are essential for safe deployment
Tuberculosis diagnosis	Uganda	Imaging-based diagnostic support	Infrastructure dependence	Contextual readiness outweighs model performance
Risk-stratified care	Mixed settings	Clinical decision support	Workflow integration	Implementation challenges dominate impact
Appropriate technology selection	Fragile settings	Tool adaptation	Misaligned assumptions	Local stakeholder engagement is critical

***Key lessons emerged:***

- **Context is critical.** Tools developed in high-resource environments often assume stable internet, interoperable data systems, maintenance capacity, and price points that do not reflect many low-resourced contexts.
- **Success is not just technical.** Implementation challenges, not model performance [21], were often the primary barriers to impact.
- **Ethical concerns persist.** From iris scanning technologies to AI-assisted triage, discussions revealed ongoing tensions around consent, privacy, surveillance, and accountability, especially in humanitarian, fragile, or other vulnerable settings.

These examples underscored a paradox: the greatest promise of AI lies in low-resource settings, yet these are also the places most structurally excluded from designing, implementing, and benefiting from it [16, 18, 22, 23]. However, recent advances in edge technology and “low-tech” native solutions are increasingly designed and utilized by low-resourced settings [24].

### Expert dialogue: Synthesis and future directions

The session ended with a moderated discussion that reinforced three cross-cutting themes: AI in global health should be co-led by public health and domain experts rather than framed as a purely technical agenda; implementation evidence matters as much as algorithmic performance; and governance, digital literacy, and infrastructure remain decisive constraints on equitable deployment. Central to this is the urgent need for enhanced AI and digital literacy across the entire health ecosystem and strong ethical governance standards.

Participants converged around five key core priorities for responsible AI deployment:

1. Establishing robust governance structures and local policymaking to protect countries from exploitation and harm;
2. Comprehensive capacity-building that extends to the entire health-technology ecosystem, including not only AI literacy but also technical and regulatory training;
3. Commitment to human- and community-centered and equity-oriented design, requiring the development and implementation of AI tools with and for intended users;
4. Use of inclusive training data beyond high-income datasets emphasizing underrepresented populations by adapting existing models or developing local ones;
5. Development of the evidence base for safe, locally responsive, and effective AI deployment in diverse global settings through rigorous, real-world evaluation, including randomized trials and cost-effectiveness studies (Table 3). Without this evidence base, AI risks scaling technical performance without demonstrable population-level benefit.

**Table 3:** What evidence is most urgently needed?

RESEARCH PRIORITY	DESCRIPTION	KEY METRIC/OUTCOME
Longitudinal performance and drift	Studies assessing model drift and performance degradation over time	Sustained accuracy, reliability, time to failure/recalibration
Cost-effectiveness analyses (CEA)	Comparing AI-enabled pathways to standard care	Incremental cost-effectiveness ratio, return on investment, total cost of ownership
Hybrid effectiveness-implementation trials	Examining adoption, retention, feasibility, fidelity, and unintended consequences of AI tools in practice	Reach, effectiveness, adoption, implementation, maintenance (RE-AIM), unintended social/workflow consequences
Comparative multi-country validation	Studies assessing the generalizability and transferability of AI models across diverse geographical and demographic populations	Cross-contextual performance stability, external validity metrics
Adaptive informed consent research	Research on informed consent models appropriate for AI-supported care, especially regarding data use and algorithmic decision-making	Patient understanding, ethical approval rates, perceived trust, and transparency

Addressing these priorities requires the development of novel, collaborative governance models that integrate expertise from public health, ethics, law, and human rights to guide cross-border AI initiatives.

These principles align with emerging global frameworks and help identify practical next steps for more equitable AI deployment in global health. Particular emphasis must be placed on LMIC leadership and co-creation in AI deployment, especially in the high-stakes field of health. Lessons from this workshop and the CUGH collaborative approach can be translated to other partnerships involving LMICs, particularly when deploying novel AI technologies.

The session also underscored the importance of coalition building, LMIC leadership, and practical guidance for implementation. Diverse participation strengthened the relevance of the discussion, while barriers such as travel costs and visa constraints highlighted how inequities in convenings can shape whose knowledge is represented. These observations reinforce the need for inclusive governance, context-specific implementation tools, hybrid-by-design convenings, and stronger mechanisms for shared decision-making.

### A call to action: Five strategic imperatives

Based on the session insights and comprehensive literature review, we propose five strategic imperatives for advancing AI in global health (Table 4):

1. ***Develop and disseminate contextualized AI governance frameworks***
  Collaborate with WHO, regional bodies, and LMIC governments to establish adaptable, enforceable AI guidelines that address ethics, consent, data ownership, accountability, and local implementation realities [4, 6, 7, 20, 25]. Our review revealed significant regulatory gaps, with LMICs particularly vulnerable to untested implementations. Contextualized governance must be tailored to local regulatory, infrastructural, and sociopolitical realities.
2. ***Invest in equitable capacity-building***
  Support digital infrastructure, local data ecosystems, and AI training initiatives that empower local actors to build, adapt, and evaluate tools. Address the digital divide that currently limits access to AI benefits in under-resourced regions, and integrate AI literacy into health professional education [10, 26, 27].
3. ***Fund implementation research and evidence generation***
  Ensure funding bodies prioritize rigorous evaluation and real-world deployment studies over purely technical performance metrics. Priority areas include pragmatic trials, cost-effectiveness, post-deployment monitoring, and comparative validation across diverse contexts.
4. ***Create open-access knowledge repositories***
  Share curricula, code, datasets, and toolkits under open science principles where appropriate to prevent duplication and promote transparency, while guarding against data exploitation and respecting data sovereignty and intellectual property rights.
5. ***Center community engagement, ethics, and equity***
  Design AI systems in dialogue with the communities they serve. Value indigenous knowledge, uphold data sovereignty, ensure benefits are reciprocal, and involve affected communities in priority setting, design, implementation, and oversight [2, 8].

**Table 4:** Strategic imperatives for equitable AI in global health and responsible actors.

STRATEGIC IMPERATIVE	PRIMARY ACTORS	SUPPORTING ACTORS	PERSISTENT GAPS
**Contextualized governance and policy**	Ministries of health, WHO, regional bodies	Legal experts, civil society	Enforcement capacity
**Capacity-building**	Universities, training institutions	Donors, NGOs	Long-term sustainability
**Evidence generation**	Researchers, implementers	Funders, policymakers	Real-world trials and outcome data
**Open knowledge**	Academic consortia, platforms	Philanthropy	Data sovereignty protections
**Community engagement**	Civil society, communities	Implementers, regulators	Power-sharing and accountability

## Conclusion

The future of AI in global health will not be determined by innovation alone but by the values, voices, and structures we embed in its development and implementation [2, 25, 28]. As we reflect on our CUGH session and landscape review, we reaffirm that equity must be more than a talking point; it must be a design feature, an implementation standard, and a governance mandate.

Our analysis across education, epidemiology, clinical medicine, and data equity reveals both tremendous potential and significant risks. Without coordinated global and local leadership, AI may reinforce inequities instead of remedying them. The path forward requires unprecedented collaboration between technologists, public health professionals, ethicists, policymakers, and, most critically, the communities AI systems aim to serve. Only through such collaboration can AI truly serve as a tool for global health equity, ensuring that technological advancement translates into more equitable health outcomes across all populations, regardless of geography, resources, or social position.
